# IRE1-XBP1 Pathway of the Unfolded Protein Response Is Required during Early Differentiation of C2C12 Myoblasts

**DOI:** 10.3390/ijms21010182

**Published:** 2019-12-26

**Authors:** Yukako Tokutake, Keita Yamada, Satoko Hayashi, Wataru Arai, Takafumi Watanabe, Shinichi Yonekura

**Affiliations:** 1 Department of Bioscience and Food Production Science, Interdisciplinary Graduate School of Science and Technology, Shinshu University, Minamiminowa, Kamiina-gun, Nagano 399-4598, Japan; yukako.tokutake.d3@tohoku.ac.jp; 2 Department of Biomedical Engineering, Interdisciplinary Graduate School of Science and Technology, Shinshu University, Minamiminowa, Kamiina-gun, Nagano 399-4598, Japan; dymdx34@gmail.com (K.Y.); hysstk_aaiaoo@yahoo.co.jp (S.H.); 18bs101c@shinshu-u.ac.jp (W.A.); 3 Laboratory of Anatomy, School of Veterinary Medicine, Rakuno Gakuen University, Ebetsu, Hokkaido 069-8501, Japan; t-watanabe@rakuno.ac.jp; 4 Department of Biomolecular Innovation, Institute for Biomedical Sciences, Interdisciplinary Cluster for Cutting Edge Research, Shinshu University, Minamiminowa, Kamiina-gun, Nagano 399-4598, Japan

**Keywords:** skeletal muscle differentiation, unfolded protein response (UPR), apoptosis, autophagy

## Abstract

In skeletal muscle, myoblast differentiation results in the formation of multinucleated myofibers. Although recent studies have shown that unfolded protein responses (UPRs) play an important role in intracellular remodeling and contribute to skeletal muscle differentiation, the involvement of IRE1–XBP1 signaling, a major UPR signaling pathway, remains unclear. This study aimed to investigate the effect of the IRE1–XBP1 pathway on skeletal muscle differentiation. In C2C12 cells, knockdown of IRE1 and XBP1 in cells remarkably suppressed differentiation. In addition, apoptosis and autophagy were dramatically enhanced in the XBP1-knockdown cells, highlighting the participation of IRE1–XBP1 in cell survival maintenance with differentiation stimuli during skeletal muscle differentiation. In myogenic cells, we demonstrated that the expression of CDK5 (cyclin-dependent kinase 5) is regulated by XBP1s, and we propose that XBP1 regulates the expression of MyoD family genes via the induction of CDK5. In conclusion, this study revealed that IRE1–XBP1 signaling plays critical roles in cell viability and the expression of differentiation-related genes in predifferentiated myoblasts and during the early differentiation phase.

## 1. Introduction

Skeletal muscle tissue is constituted by multinucleated myofibers formed as a result of mononuclear myoblasts fused to each other. The process of myofiber formation during embryonic development and muscle regeneration (i.e., when the muscles are damaged) depends on muscle stem cells called satellite cells. Also known as myogenic differentiation, this process is driven by extracellular environmental stimuli, which include various cytokines. Myogenic differentiation is divided into several different stages [1]. In their mononuclear stages, MyoD and Myf5 are involved in the determination of myoblasts, maintenance of cell proliferation, and induction of subsequent gene expression [2,3]. After differentiation induction, the myoblasts undergo cell cycle arrest, and the gene expression of myogenin and Mef2c is induced, leading to myoblast cell fusion and terminal differentiation [4,5]. Although previous studies have identified factors and signaling pathways essential for the late stage of myogenic differentiation to control cell fusion, myofiber formation, and terminal differentiation, the early stage just before and after initial differentiation remains to be characterized.

In the early stages of myogenic differentiation, remodeling of the intracellular organelles in myoblasts is facilitated in response to increasing intracellular energetic demands and homeostatic changes in calcium (Ca^2+^) fluxes [6,7]. Mitochondrial clearance and subsequent mitochondrial neogenesis are induced by an increase in the production of ATP required by cells after the induction of differentiation to form myofibers [7]. Similarly, transient Ca^2+^ depletion occurs in the endoplasmic reticulum (ER) of myoblasts undergoing differentiation due to alterations in the intracellular Ca^2+^ fluxes. Nakanishi et al. reported that Ca^2+^ depletion increases stress in the ER lumen of myoblasts. Although chronic ER stress can cause pathological alterations, including dystrophy, in muscles [8], previous findings have reported a positive role of ER stress in regulating skeletal muscle differentiation. As demonstrated in a study wherein myogenic differentiation is suppressed concomitantly with mitigation of ER stress when Ca^2+^ fluxes are inhibited, alterations in intracellular Ca^2+^ flux are necessary to promote ER development, which is in turn essential for myogenesis [6]. Furthermore, transient ER stress in myoblasts in vitro dramatically improves myotube (i.e., immature myofiber) formation [9]. Taken together, intracellular stress that increases in several organelles owing to alteration of functional demands is an important factor regulating myogenic differentiation. These findings suggest that the physiological mild ER stress, which occurs during muscle cell differentiation, improves the myogenic differentiation efficiency. Consequently, elucidating the function of ER stress response signals induced by mild ER stress is essential for understanding skeletal myogenic differentiation.

The unfolded protein response (UPR) mechanism is an integrated signaling pathway that allows cells to alleviate ER stress, increase their protein-folding efficacy, or trigger apoptosis [10,11]. UPRs are initiated via the activation of the three major ER transmembrane sensor proteins IRE1α (inositol-requiring enzyme 1), PERK (protein kinase RNA-like ER kinase), and ATF6 (activating transcription factor 6) [12]. In the ER lumen, each sensor protein interacts with BiP/GRP78 to suppress UPR signal activation under unstressed conditions. When ER stress occurs, UPR activation by the sensor proteins induces self-oligomerization or translocation of GRP78 following its dissociation from BiP, and each inducer activates downstream factors to remove unfolded proteins from the ER lumen and to achieve ER homeostasis [10,13]. The sensor protein IRE1 is activated by oligomerization and autophosphorylation [14]. The endoribonuclease (RNase) activity of activated IRE1 splices the 26 nucleotides of the pre-XBP1(X-box binding protein 1) mRNA intron [15,16]. The mature form of XBP1 (spliced XBP1:XBP1s) transactivates gene expression related to protein folding, ER quality control, and endoplasmic reticulum-associated proteolysis [17,18]. PERK induces the transcription factor ATF4 (activating transcription factor 4) by inactivating the translation initiation factor eIF2α (eukaryotic initiation factor 2) [19]. ATF4 induces CHOP (C/EBP homologous protein) gene expression. Both ATF4 and CHOP act as transcription factors for recovering from ER stress and inducing apoptotic cell death [20]. ATF6 moves from the ER to the Golgi apparatus to be cleaved by site-1 protease [21]. The cleaved ATF6 (N) then acts in combination with XBP1s to increase UPR-related gene transcription to alleviate ER stress [18]. UPR transcription factors were recently shown to play a role in not only dissolving ER stress but also the differentiation of various cell types [22,23,24,25,26]. In skeletal muscles, PERK and ATF6 play crucial roles in myogenic differentiation [27,28]. In particular, in vitro analysis showed that PERK activity increases when apoptosis is induced within 24 h after differentiation initiation and that PERK deficiency decreases the number of viable myoblasts [28].

In a previous study, we found that C2C12 mouse skeletal muscle cells overexpressing the P56S-missense mutated vesicle-associated membrane protein/synaptobrevin-associated membrane protein (VAPB), which is the causative gene of familial amyotrophic lateral sclerosis (ALS) [29], exhibit marked myotube formation disruption and IRE1 activation inhibition [30]; this suggests that IRE1 positively controls myogenic differentiation as well as other UPR-sensor factors. Acosta et al. reported that overexpression of spliced XBP1 suppresses C2C12 myogenic differentiation [31]. However, it only assessed the effects of transient overexpression, and it is still unclear whether the IRE1–XBP1 axis is essential for myogenic differentiation [31]. Therefore, the present study aimed to elucidate how the IRE1–XBP1 axis is involved in myogenic differentiation.

## 2. Results

### 2.1. IRE1–XBP1 Is Essential for the Myotube Formation in C2C12 Differentiation

**Figure 1:**
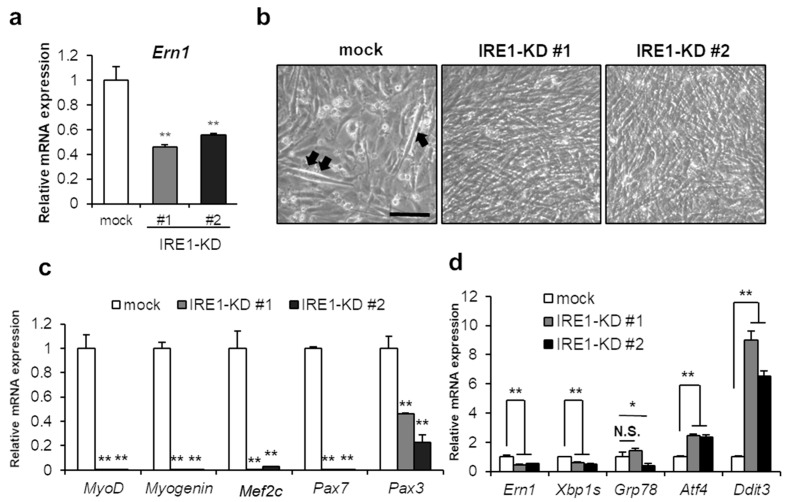
IRE1 is required in C2C12 differentiation. (**a**) IRE1-knockdown cell lines #1, #2, and mock cells were evaluated for the expression of IRE1/*Ern1* mRNA. Results are means + SEM (three biological replicates). ** *p* < 0.01. (**b**) Differentiation was induced in IRE1-knockdown cell lines and mock cells. After 48 h, cells were observed under phase contrast. Black arrows show the immature myotubes. Scale bar = 100 µm. (**c**,**d**) Cells were harvested after differentiation induction at 48 h. mRNA expression of myogenic factors (**c**) and UPR relative factors (**d**) was analyzed by qPCR. Results are means + SEM (three biological replicates). * *p* < 0.05, ** *p* < 0.01.

First, we assessed whether IRE1-XBP1 was involved in myogenic differentiation. To determine the impact of IRE1 on skeletal muscle differentiation, we generated IRE1-knockdown C2C12 cell lines (IRE1-KD #1, #2) that stably expressed IRE1 shRNA. IRE1/*Ern1* deficiency was confirmed by mRNA level in two cell lines (Figure 1a). Seventy-two hours after differentiation induction, IRE1-KD cells in both lines were detached and could not be cultured anymore. At 48 h after differentiation induction, no myotubes were formed in IRE1-KD cells whereas mock cells formed immature myotubes (Figure 1b). Furthermore, expression of myogenesis-related genes, also called MRFs, was markedly declined including not only *Myogenin* and *Mef2c* (essential for skeletal muscle differentiation), but also *MyoD, Pax7*, and *Pax3*, involved in the specification and proliferation of muscle progenitor cells (Figure 1c). Interestingly, the expression of the PERK pathway downstream factors, *Atf4* and CHOP/*Ddit3*, was remarkably increased in the IRE1-KD cells (Figure 1d). Nevertheless, the expression of *Grp78*, a UPR-related molecular chaperone, did not change when compared with mock cells (Figure 1d). Taken together, the knockdown of IRE1 remarkably repressed myogenic differentiation.

**Figure 2:**
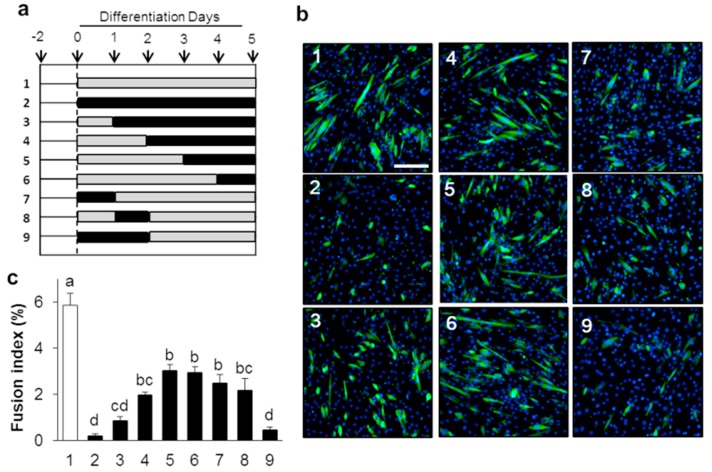
IRE1 ribonuclease activity is required in early phase of C2C12 differentiation. (**a**) Differentiation was induced in the presence or absence of IRE1 RNase inhibitor, STF-083010 (60 µM; black bars) or DMSO (gray bars) for various time intervals as indicated. (**b**) Identification of critical time period for inhibitory effect of IRE1 activity on C2C12 differentiation. Scale bar = 200 µm. (**c**) Fusion index of STF-083010- or DMSO-treated cells. Results are mean + SEM (three biological replicates). The different letters denote significant differences between groups at *p* < 0.05 by Tukey’s HSD test.

Next, we investigated the impact of IRE1 RNase activity inhibition on myogenic differentiation. STF-083010, an RNase-specific activity inhibitor of IRE1 [32], was supplemented to the cells until 5 days after differentiation induction (Figure 2a,b). As compared with control cells, MHC-positive myotubes were markedly reduced by STF-083010 treatment (Figure 2b-1,b-2). Importantly, an inhibitory effect of myotube formation was observed in cells supplemented with STF-083010 only for 0–48 h after the differentiation induction (Figure 2b-9). Fusion index also demonstrated that inhibitory effects of cell fusion were equally low in group 2 and group 9 (Figure 2c).

**Figure 3:**
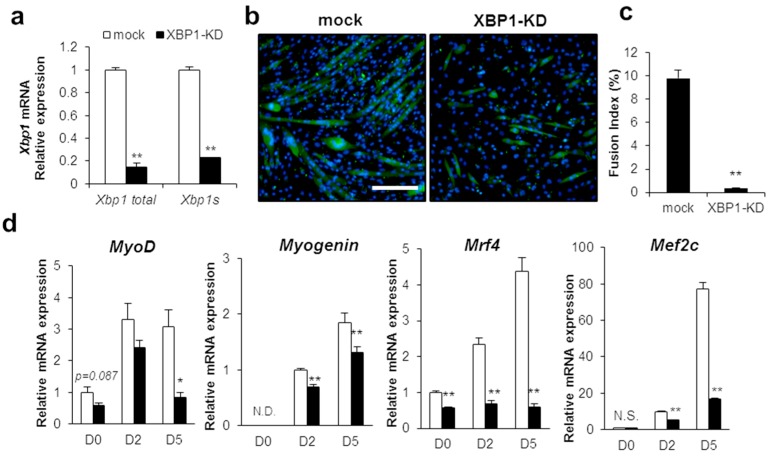
XBP1 is required for C2C12 differentiation. (**a**) mRNA expression of *Xbp1* and *spliced Xbp1* were compared between XBP1-knockdown cells (XBP1-KD) and mock cells. Results are means + SEM (three biological replicates). Student’s *t*-test. ** *p* < 0.01. (**b**) XBP1-knockdown cells and mock cells were induced to differentiate until day 5. Cells were observed for immunofluorescent staining with anti-MHC antibody. Scale bar = 200 µm. (**c**) Fusion index of mock or XBP1-KD cells. Results are mean + SEM (three biological replicates). Student’s *t*-test. ** *p* < 0.01. (**d**) Cells were harvested on the indicated day. mRNA expression of each myogenic factor was analyzed by qPCR. Results are means + SEM (three biological replicates). Student’s *t*-test. * *p* < 0.05, ** *p* < 0.01.

To determine the impact of XBP1 deficiency on differentiation, XBP1 shRNA was stably expressed in C2C12 cells. The XBP1-knockdown (XBP1-KD) cell line was tested to confirm that not only XBP1s but also prespliced mRNA expression was suppressed to 20% of the mock level (Figure 3a). In XBP1-KD cells, myotube formation was inhibited at 5 days after differentiation induction (Figure 3b,c). Moreover, MRFs (*MyoD, Myogenin, Mrf4,* and *Mef2c*) expression was significantly repressed with the differentiation (Figure 3d). After differentiation induction, the expression of *Myogenin* and *Mef2c*, a marker gene for late differentiation, was lower than control (Figure 3d). In addition, *Mrf4* expression was remarkably suppressed in the undifferentiated cells as well as in the cells after differentiation induction (Figure 3d). These results suggest that inhibition of the IRE1 RNase activity and XBP1 markedly represses differentiation. In particular, IRE1 RNase activity is essential from the myoblast to early differentiation stage.

### 2.2. XBP1 Deficiency Perturbs Apoptosis and Autophagy during Early Myogenic Differentiation

**Figure 4:**
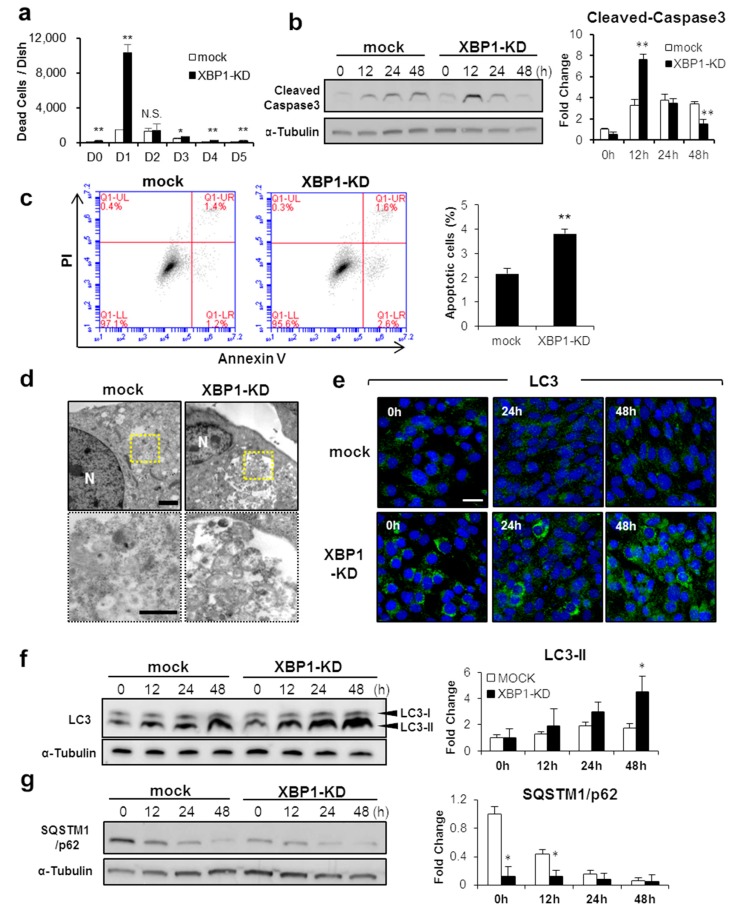
XBP1 protects myoblasts from apoptosis and autophagy during myogenesis. (**a**) Differentiation was induced in XBP1-knockdown and mock cells. After culturing, the number of dead cells was counted at the indicated time. Results are means + SEM (three biological replicates). Student’s *t*-test. * *p* < 0.05, ** *p* < 0.01. (**b**) Expression of cleaved caspase-3 was detected by Western blotting. Results are means + SEM (*n* = 3). Student’s *t*-test. ** *p* < 0.01. (**c**) Flow cytometry was used to analyze annexin V-FITC/PI double staining of mock and XBP1-KD cells undergoing apoptosis. Results are means + SEM (three biological replicates). Student’s *t*-test. ** *p* < 0.01. (**d**) Representative high-magnification TEM images. XBP1-knockdown cells were observed for the formation of autophagosomes. N: nucleus. Lower images are enlarged views of the indicated area in upper images (yellow box). Scale bars = 2 µm (upper), 1 µm (lower). (**e**) Differentiation was induced in XBP1-knockown and mock cells and the cell lines cultured for 48 h. Cells were immunostained with anti-LC3 antibody (green). DAPI (blue) indicates nuclei. Scale bar = 50 µm. (**f**,**g**) Expression of LC3 and SQSTM1/p62 protein levels were analyzed by Western blotting. Results are means + SEM (three biological replicates). Student’s *t*-test. * *p* < 0.05.

In myogenic differentiation, apoptotic cell death is induced both in vivo and in vitro [33]. Apoptosis is essential for myogenic differentiation, and inhibition of apoptosis results in suppression of myogenesis [27,34,35]. Since XBP1s is an important factor of early-stage myogenic differentiation, we tested whether XBP1s is involved in inducing apoptosis. In control C2C12 cells, the number of dead cells was increased and peaked at 2 days after differentiation induction, before gradually decreasing (Figure 4a). On the other hand, a significant increase of dead cells was observed in XBP1-KD cells compared to control cells throughout the differentiation process. Interestingly, the number of dead cells was markedly increased about 7-fold at day 1 after induction of differentiation and decreased at day 2 (Figure 4a). In control C2C12 cells, the expression of cleaved caspase-3 increased from 12 h after the differentiation induction and was then maintained at a steady level (Figure 4b). In XBP1-KD cells, however, the expression rapidly increased at 12 h after the induction of differentiation and decreased to a level lower than the level of control cells at 48 h (Figure 4b). Expression patterns of cleaved caspase-3 in XBP1-KD cells were consistent with increasing dead cell number (Figure 4a). Similarly, annexin V-positive apoptotic cells were increased in XBP1-KD cells at 12 h after the induction of differentiation (Figure 4c). These results indicate that XBP1-KD cells undergo rapid apoptotic cell death immediately after induction of differentiation.

In parallel with apoptotic cell death, autophagy is induced during early myogenesis for intracellular organelles clearance and to protect from cell death [7,36]. To test the impact of XBP1 deficiency on autophagy induction, the formation of autophagosomes was confirmed by TEM. At 48 h of differentiation induction, we observed autophagosome formation in the form of vesicles surrounding vacuolar objects in both mock and XBP1-KD cells (Figure 4d). However, greater autophagosome accumulation was confirmed in XBP1-KD cells (Figure 4d). The expression of LC3, a typical autophagy-related protein, also increased in differentiated cells and LC3 puncta, reflecting autophagosome formation, and was more abundant in XBP1-KD cells than in control cells (Figure 4e). The expression of autophagosomal membrane-bound LC3 (LC3-II) was significantly increased compared with controls at 48 h (Figure 4f). Furthermore, the expression of p62/SQSTM1, a selective substrate that serves as an adaptor protein in autophagosomes, was underrepresented throughout the differentiation process in XBP1-KD cells (Figure 4g). These results demonstrate that XBP1 deficiency causes increased apoptotic cell death and promotes autophagy induction during early skeletal muscle differentiation.

### 2.3. CDK5 Is a Transcriptional Target Gene of XBP1s

**Figure 5:**
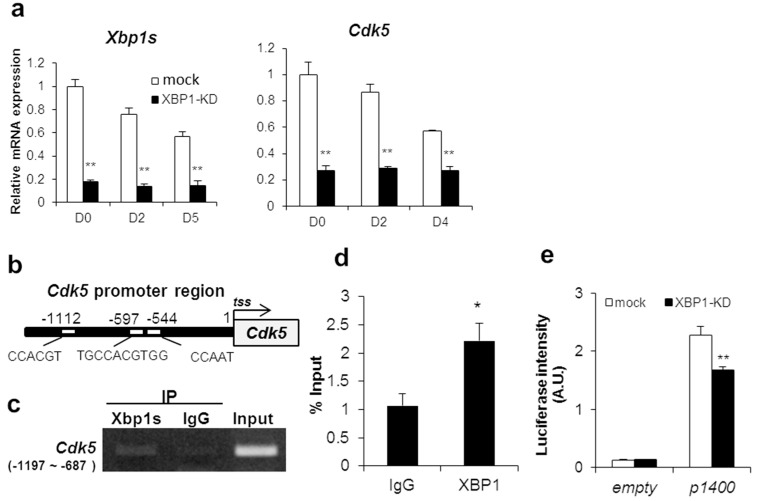
CDK5 is a downstream target of IRE1-XBP1 in C2C12 cells. (**a**) mRNA expression of *Xbp1* and *Cdk5* during C2C12 differentiation. Results are means + SEM (three biological replicates). Student’s *t*-test. ** *p* < 0.01. (**b**) An illustration of the predicted mouse *Cdk5* promoter region. The region upstream of the *Cdk5* gene comprises three XBP1-binding domains including CCAAT at −544 bp, TGCCACGTGG at −597 bp, and CCACGT at −1112 bp from the transcription start site. (**c**,**d**) Chromatin immunoprecipitation assay using a C2C12 genomic sample. Input DNA = positive control. Rabbit IgG was used as negative control ChIP (**c**). ChIP assay was performed by quantitative PCR analysis (**d**). Results are means + SEM (three biological replicates). Student’s *t*-test. * *p* < 0.05. (**e**) XBP1-knockdown and mock cells were transfected with the vector containing the *Cdk5* promoter construct (*p*1400) or an empty vector. *Cdk5* promoter activity was assessed by luciferase assay. Results are means + SEM (three biological replicates). Student’s *t*-test. ** *p* < 0.01.

Since IRE1-XBP1s positively regulates myogenic differentiation, we explored downstream factors of XBP1s in C2C12 myogenic differentiation. Acosta et al. have comprehensively analyzed promoter regions by ChIP-on-chip analysis and have found several target genes possibly regulated by XBP1s in C2C12 myotube cells [31]. We focused on the promoter of cyclin-dependent kinase 5 (CDK5), a gene previously shown to be involved in myogenic cell differentiation and patterning [37]. Although Cdk5 belongs to the cyclin-dependent kinase family, CDK5 is not activated by cyclins, nor does it contribute to cellular growth regulation like other members of the CDK family [38]. In addition, *Cdk5* gene expression in C2C12 cells was highest in undifferentiated cells and decreased after differentiation induction similar to the Xbp1s expression pattern (Figure 5a). Also, *Cdk5* gene expression was significantly suppressed in XBP1-knockdown cells (Figure 5a).

Therefore, we investigated whether *Cdk5* is a transcriptional target gene of XBP1s. We identified three XBP1 binding domains within 1200 bp upstream from the transcription start site in murine *Cdk5*: CAAT-box (CCAAT) [18], ACGT core (CCACGT) [31], and the single-nucleotide insertion of UPRE-like domain (TGCACGTGG) [16] (Figure 5b). The ChIP assay showed that XBP1 binds to the region from −1197 to −687 bp upstream of *tss* (Figure 5c,d). To elucidate whether XBP1 regulates *Cdk5* transcriptional activity via this gene region, the *Cdk5* predicted promoter region was cloned and a luciferase reporter assay was performed. Transcriptional activity in XBP1-knockdown cells was suppressed by approximately 30% compared to control (Figure 5e). Therefore, XBP1s is confirmed as a transcriptional regulator of CDK5. Taken together, these results indicate that XBP1s is involved in myogenic differentiation inducing MRFs through CDK5.

## 3. Discussion

Myogenic differentiation involves ER stress and UPR signals. The ER stress-specific apoptosis initiation factor caspase 12 is transiently activated in muscle tissue during mouse embryonic developmental period (i.e., around E13.5) [27,39]. This result strongly suggests that ER stress plays an important role in triggering apoptosis associated with muscle development. Also, the UPR-sensor molecules ATF6 and PERK have been reported to be essential factors for skeletal muscle differentiation [27,28]. Because both of these factors are involved in apoptosis induction and cell survival during myogenic differentiation, it has been suggested that UPR plays an important role in the early stage of myogenesis. Our current study demonstrates that the UPR factors IRE1 and XBP1s are indispensable for myogenic differentiation.

In IRE1-knockdown cells, the expression of myoblast specification and proliferation genes, including MyoD and Pax-family members, were markedly suppressed. The overexpression of dominant-negative constructs of Pax3 and Pax7 was previously shown to prevent myogenic differentiation and was associated with cell morphology alteration in C2C12 cells [40]. Thus, the marked suppression of differentiation observed in IRE1-knockdown cells may be due to the inhibition of the function of Pax-family members. In addition, the expressions of the PERK pathway downstream factors ATF4 and CHOP were increased in IRE1-knockdown cells, and no changes were observed in the expressions of ATF4 and CHOP in the XBP1-knockdown cells (Supplementary Figure S1), suggesting that PERK signaling is not dependent on XBP1s. Thus, IRE1 deficiency might induce a compensatory activation of the PERK pathway and the imbalance of UPR-sensor proteins activity might also play critical roles as negative mediators of myogenic gene expression during myogenesis.

Our observation revealed that XBP1 is an essential factor for myogenic differentiation. Recently, Jheng et al. reported that knockdown of XBP1 by siRNA transfection after differentiation onset affected cellular development at a late stage during myogenic differentiation [41]. However, the expression of MyoD and MRF4, which are involved in the process of myoblast specification, is already lower in XBP1-knockdown cells than in control cells [1,42]. Moreover, our study concerning the inhibitors of IRE1 activity and additional analyses clearly demonstrate that IRE1–XBP1 is rather involved in the early stages of differentiation. These results suggest that the involvement of XBP1 is more important in the early rather than the late differentiation stage. Acosta et al. demonstrated that the overexpression of XBP1s suppresses muscle differentiation by inducing the MyoD repressor Mist1 that binds MyoD to inhibit its transactivation domain [31]. A putative “ER stress control checkpoint” functioning early in lineage determination has been proposed. During myogenesis, surpassing a threshold level of XBP1s would promote Mist1 activation, thereby impeding differentiation and restoring homeostasis under excessive stress. Our study indicates that lowering IRE1–XBP1 activation also affects progression to differentiation at the early determination stage; therefore, our data support the hypothesis of Acosta et al.

As observed in the XBP1-knockout cre–pax7 mouse, in the satellite cell-mediated muscle regeneration process, XBP1 deletion did not affect muscle regeneration [28]. We hypothesized that different myogenic factors contribute to embryonic myogenesis and muscle regeneration mediated by satellite cells. Indeed, MRF4 is involved in myoblast specification during the embryonic stage but not in the muscle regeneration process [43,44]. Therefore, XBP1 deletion has either no significant impact or is compensated by other factors in satellite cell-mediated muscle regeneration. Further analysis is required to discriminate between these possibilities.

Previously, apoptosis and autophagy induced in the early stages of muscle differentiation were reported as essential for organelle remodeling and myotube formation [7,35]; however, excessive apoptosis and autophagy negatively affect cell survival and differentiation. XBP1 deficiency abruptly increases apoptosis and autophagy, suggesting that XBP1 functions in moderating these events. Of note, we found that AMPK activation is increased in XBP1-knockdown cells. AMPK activation is enhanced by intracellular ATP depletion and is also involved in autophagy induction [45,46]. Based on the concomitant reduction in XBP1 expression and myogenic differentiation, XBP1s might be involved in myogenic cell adaptation and viability by switching to intracellular energy metabolism when cells respond to differentiation induction.

The present study revealed that XBP1 induces CDK5 expression. CDK5 is intimately involved in skeletal muscle development, exhibiting a marked increase during early myogenesis with a peak between 36 and 48 h of differentiation in vitro [47]. In *xenopus* embryos, the expression of both MyoD and MRF4 is markedly reduced by a dominant-negative form of CDK5, and somatic muscle patterning is inhibited [37]. Indeed, because *Cdk5*, *MyoD*, and *Mrf4* expression was also significantly reduced in XBP1-knockdown cells, CDK5 likely contributes to the XBP1-knockdown phenotype. However, XBP1 only partially induced *Cdk5* transcriptional expression because only 30% of the reporter activity was suppressed by XBP1 deficiency (Figure 5e). A transcriptional regulator of Cdk5 as well as XBP1 may be existent in myoblasts. Although not all genes regulated by XBP1s have been identified, Acosta et al. previously identified at least 148 candidate genes regulated by XBP1s in differentiated C2C12 cells [31]. Thus, there might be factors among these that are transcriptionally regulated by XBP1 and involved in differentiation regulation.

Taken together, the present report revealed that both IRE1 and XBP1 play critical roles in myogenic differentiation. In particular, XBP1s are involved in apoptosis and autophagy occurring in the first 24 h after differentiation induction. Furthermore, the expression of the myogenic regulation factors MyoD and MRF4 is partially mediated by CDK5, a transcriptional target of XBP1s in myoblast cells. Thus, the IRE1–XBP1 pathway contributes to myogenic differentiation as a key regulator from the undifferentiated to early differentiated stages of myogenesis. UPR participates positively and negatively in myogenic differentiation. Whereas the mild stress condition caused in physiologically is driven by UPR-mediated muscle tissue development, when excessive ER stress occurs, inhibition of myogenesis protects myoblasts from apoptosis. The specificity of the UPR function between “for recovering homeostasis” and “for adapting to the external environment” remains to be elucidated. Our findings contribute to understanding how the ER stress positive effect is mediated by the UPR pathway during skeletal muscle development.

## 4. Materials and Methods

### 4.1. Reagents and Antibodies

Dulbecco’s modified Eagle medium (DMEM) was purchased from Thermo Fisher Scientific, Inc. (Waltham, MA, USA). Fetal bovine serum (FBS) was purchased from Microbiological Associates (Bethesda, MD, USA). Horse serum (HS) was purchased from Thermo Fisher Scientific, Inc. SDS-PAGE gels and PVDF membranes were purchased from Bio-Rad (Hercules, CA, USA). STF-083010 and roscovitine were purchased from SIGMA (Saint Louis, MO, USA). All other compounds were purchased from Wako Pure Chemical Company (Osaka, Japan). Immunoblotting analysis was performed using the following antibodies: anti-α-tubulin, rabbit polyclonal antibody (MBL, Nagoya, Japan), anti-cleaved caspase-3, anti-LC3B, anti-SQSTM1/p62 rabbit polyclonal antibody (Cell Signaling Technology, Beverly, MA, USA), anti-rabbit or anti-mouse IgG HRP-linked whole Ab (GE Healthcare, Chicago, IL, USA).

### 4.2. Cell Culture

C2C12 mouse myoblasts were obtained from DS Pharma Biomedical (Osaka, Japan). The cells were cultured as described previously [30]. Briefly, the cells were cultured in growth medium (DMEM/FBS 10%) under 5% CO_2_ at 37 °C; differentiation was induced by changing the medium (DMEM/HS 2%) once the cells reached confluency. For transient transfection, the cells were transfected with Lipofectamine 2000 (Thermo Fisher Scientific), following the manufacturer’s protocol. To quantify the dead cells, the culture supernatants containing dead cells were collected after trypsinization and centrifuged at 500× *g*. The dead cells were resuspended in trypan blue and counted in triplicates per sample using a hemocytometer.

### 4.3. Establishment of shIRE1- and shXBP1- Transduced Cells

IRE1α shRNA plasmid was purchased from Santa Cruz Biotechnology, Inc. (Dallas, TX, USA). PLKO-puro with mouse XBP1 shRNA plasmid was received as a gift from Dr. Ann-Hwee Lee, Ph.D. (Senior Staff Scientist at Regeneron Pharmaceuticals, Inc. Tarrytown, NY, USA). To establish stable cell lines expressing the IRE1–shRNA constructs, C2C12 cells were transfected using Lipofectamine 2000 (Thermo Fisher Scientific). The transfected cells were plated at low density and then selected using 3 μg/mL puromycin (Thermo Fisher Scientific); further, clonal colonies were isolated. IRE1 expression levels were analyzed by immunoblotting analysis. To establish shXBP1-transduced cells, lentiviral constructs were established for use with the Lenti-X Expression System (TaKaRa Bio Inc., Shiga, Japan). HEK293T cells were transfected with vectors using Lipofectamine 2000. After 48 h, the virus-containing supernatant was harvested. Transduction of C2C12 was performed with shIRE1 lentivirus (multiplicity of infection:10) in the presence of 5 µg/mL polybrene, and cells were selected with 3 μg/mL puromycin (Thermo Fisher Scientific) for five days. Single colonies were picked under light microscopy at the fifth passage.

### 4.4. Quantification of Myotube Formation

The differentiation potential of the myoblasts, known as the fusion index, was evaluated as a percentage of the number of nuclei contained within mouse anti-Myosin 4 (MF20)-positive myotubes per total number of nuclei. At least 5000 nuclei from several random fields were counted for group (three biological replicates).

### 4.5. Apoptosis Analysis by Flow Cytometry

Apoptosis was determined using annexin-V/PI double staining with an Apoptotic/Necrotic Cells Detection Kit (TaKaRa Bio). Cells were cultured in six-well plates and differentiation was induced (approximately 1 × 10^6^ cells/well). After 12 h, cells were collected by trypsinization, washed twice with ice-cold PBS, and then resuspended in binding buffer. Then, cells were stained with FITC-annexin V and ethidium homodimer III (EthD-III) for 15 min in the dark. Cells were analyzed using the BD Accuri C6 and BD CellQuest Pro software programs (BD Biosciences Pharmingen, San Diego, CA, USA).

### 4.6. Transmission Electron Microscopy (TEM)

Cells were prefixed in 2.5% glutaraldehyde and subsequently fixed in 0.1 M sodium cacodylate buffer (pH 7.4) containing 1% osmium tetroxide for 1 h at 4 °C. The fixed cells were then stained en bloc, postfixed in 1% osmium tetroxide, dehydrated through a graded ethanol series, and embedded in epoxy resin. Ultrathin sections (∼90 nm) were then cut using Ultracut UCT (Leica Microsystems, Wetzlar, Germany), stained with aqueous lead citrate and uranyl acetate, and examined using TEM (JEM 1400; JEOL, Tokyo, Japan) at an acceleration voltage of 75 kV.

### 4.7. Immunocytochemistry

Immunocytochemistry was performed as described previously [30]. Briefly, cells were washed with PBS, fixed with 4% paraformaldehyde in PBS, and blocked with 10% goat serum in PBS with 0.01% Triton-X 100. Then, the cells were incubated with mouse anti-Myosin 4 (MF20) monoclonal primary antibody (Thermo Fisher Scientific) (1:50) for 2 h at room temperature or with anti-LC3 antibody overnight at 4 °C. The cells were then incubated with Alexa-Fluor^®^ 488-conjugated goat anti-mouse/rabbit secondary antibody (1:200) IgG (H + L) (Thermo Fisher Scientific) for 1 h. The cell nuclei were stained with DAPI (Thermo Fisher Scientific) and observed under a confocal laser-scanning microscope (FLUOVIEW FV-1000; Olympus Optical Co Ltd, Tokyo, Japan) or the EVOS^®^ FL Auto Cell Imaging System (Thermo Fisher Scientific).

### 4.8. Immunoblotting

Western blotting was performed to detect cleaved caspase-3, LC3, p62/SQSTM1, and α-tubulin as previously described. Briefly, the cells were harvested and lysed in RIPA lysis buffer [50 mM Tris-HCl (pH 7.4) containing 1% NP-40, 0.25% sodium deoxycholate, 0.1% SDS, 150 mm NaCl, 1 mM EDTA, and 1× protease inhibitor cocktail (Nacalai Tesque, Inc., Kyoto, Japan)] to prepare protein extracts. The samples were size-fractionated using SDS-PAGE and subsequently immunoblotted. The blots were blocked with 4% nonfat dried milk in TBS + 0.01% Tween 20 and then incubated with primary followed by secondary antibodies in blocking buffer. Labeled proteins were visualized using the ECL Prime Western Blotting Detection Reagent kit (GE Healthcare).

### 4.9. ChIP Assay

For chromatin immunoprecipitation analysis, the C2C12 cells were cultured for 48 h and fixed in 1% paraformaldehyde/PBS for 10 min at room temperature. Chromatin shearing and immunoprecipitation were performed using the Pierce Magnetic ChIP kit (Thermo Fisher Scientific), following the manufacturer’s protocol. The immunoprecipitated DNA fragments were used as templates for amplification with PCR performed using the following primers for Cdk5-ChIP: forward, 5′-aaagacccttgcgtgtcatc-3′ (approximately −1223 through −1204; +1 indicates the transcription start site of each gene) and reverse, 5′-ttcgagacgataaaatgggc-3′ (approximately −732 through −713). The PCR products were separated on a 3% agarose gel and visualized.

### 4.10. Luciferase Assay

The pGL3 basic vector (Promega, Madison, WI, USA) was constructed with the promoter of mouse Cdk5, corresponding to the region of −1417 to +20 bp with respect to the putative transcription start site. The pRL-TK vector was used to normalize transfections. Mock and XBP1-KD cell lines were seeded at 0.7 × 10^4^ cells/well into 96-well plates (Coster, Cambridge, MA, USA) and transiently cotransfected with two vectors. Transcriptional activity was analyzed using the Dual-Glo Luciferase Assay System (Promega), following the manufacturer’s protocol. Firefly luciferase activity was normalized by Renilla luciferase activity. All experiments were independently performed in triplicates.

### 4.11. RNA Extraction, Real-Time PCR Analysis

Total RNA was isolated from C2C12 cells (for real-time PCR) using the TRIzol reagent (Thermo Fisher Scientific), following the manufacturer’s instructions. For real-time PCR, the samples were processed for cDNA synthesis using a qPCR RT Master Mix with gDNA Remover (TOYOBO, Osaka, Japan). Quantitative real-time PCR was performed using SYBR Premix Ex Taq TM II (TaKaRa Bio). Relative expression was normalized to TATA-binding protein (*Tbp*) gene expression. The sequences of the primer used are as follows: *Tbp* Forward: 5′-cattctcaaactctgaccactgcac-3′, Reverse: 5′-cagccaagattcacggtagatacaa-3′; *Xbp1(s)* Forward: 5′-tgagaaccaggagttaagaacacg-3′, Reverse: 5′-cctgcacctgctgcggac-3′; *Xbp1(total)* Forward: 5′-acatcttcccatggactctg-3′, Reverse: 5′-taggtccttctgggtagacc-3′; IRE1/*Ern1* Forward: 5′-acgaaggcctgacgaaactt-3′, Reverse: 5′-atctgaacttcggcatgggg-3′; *Grp78* Forward: 5′-gtcttcaatgtccgcatcctg-3′, Reverse: 5′-gaaaggatggttaatgatgctgag-3′; *Atf4* Forward: 5′-gagcttcctgaacagcgaagtg-3′, Reverse: 5′-tggccacctccagatagtcatc-3′; CHOP/*Ddit3* Forward: 5′-cctagcttggctgacagagg-3′, Reverse: 5’-ctgctccttctccttcatgc-3′; *MyoD* Forward: 5′-tgagcaaagtgaatgaggccttc-3′, Reverse: 5′-tgcagaccttcgatgtagcggat-3′; *Myogenin* Forward: 5′-tacgtccatcgtggac agcat-3′, Reverse: 5′-tcagctaaattccctcgctgg-3′; *Mrf4* Forward: 5′-ccaagtgttcggatcattcca-3′, Reverse: 5′-gctgaggcatccacgtttg-3′; *Mef2c* Forward: 5′-tggcagcaagaacacgatgc-3′, Reverse: 5′-aggagttgctacggaaaccac-3′; *Cdk5* Forward: 5′-ggcaatgatgtgatgaccag-3′, Reverse: 5′-ggtagggcttatagtctggcag-3′. All quantitative RT-PCRs were performed using the Thermal Cycler Dice Real-Time System (TaKaRa Bio) based on the ΔΔCt method. All data were analyzed as recommended by the manufacturer.

### 4.12. Statistical Analysis

Based on three repeats in each experimental group, all data are represented as mean ± standard error of the mean. Statistical significance was determined using the Student’s *t*-test for comparisons between two samples or ANOVA with post hoc Tukey–Kramer’s honestly significant difference test for multiple comparisons. *p* < 0.05 was considered statistically significant. 

## 5. Conclusions

This study aimed to investigate the effect of the IRE1–XBP1 pathway on skeletal muscle differentiation using C2C12 cell lines. Results with knockdown cell lines or chemical inhibitor suggest that the IRE1–XBP axis is important for the cell survival and myogenic genes expression in C2C12. We also found that Cdk5 is a direct target of XBP1.

## Supplementary Materials

The following is available online at https://www.mdpi.com/1422-0067/21/1/182/s1.

## Abbreviations

UPR	Unfolded Protein Response
IRE1	Inositol-requiring Enzyme 1
XBP1	X-box Binding Protein 1
PERK	Protein Kinase R-like Endoplasmic Reticulum Kinase
CHOP	C/EBP Homologous Protein
ATF4	Activating Transcription Factor 4
MyoD	Myogenic Differentiation 1
Myf5	Myogenic Factor 5
MRF4	Myogenic Regulatory Factor 4
MEF2C	Myocyte Enhancer Factor 2
CDK5	Cyclin-dependent Kinase 5
