# Supplementary material for: IRE1-XBP1 Pathway of the Unfolded Protein Response Is Required during Early Differentiation of C2C12 Myoblasts
Source: Int J Mol Sci. 2019 Dec 26;21(1):182. doi: 10.3390/ijms21010182 (PMC6981822; doi:10.3390/ijms21010182)
Supplement: Supplementary file 1 [file ijms-21-00182-s001.pdf]

Supplementary Materials:

IRE1-XBP1 Pathway of the Unfolded Protein Response Is Required during Early Differentiation of C2C12 Myoblasts

Yukako Tokutake [1,†], Keita Yamada [2], Satoko Hayashi [2], Wataru Arai [2], Takafumi Watanabe [3] and Shinichi Yonekura [1,2,4,*]

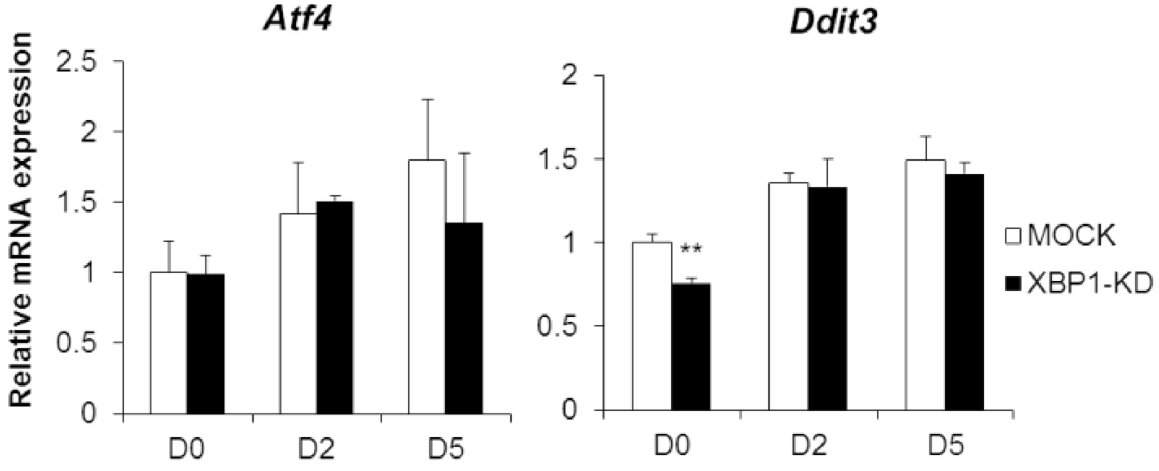

Figure S1. Cells were harvested on the indicated day. mRNA expression of each myogenic factor was analyzed by qPCR. Results are means + SEM (three biological replicates). Student's *t-test*. ** $p < 0.01$.
